# Effect of Seasonal Variation on the Incidence of Gallbladder Stone Complications

**DOI:** 10.7759/cureus.83964

**Published:** 2025-05-12

**Authors:** Sultan H Alsaigh, Abdulhakeem A Aloqla, Faisal O Alzaaqi, Asma A Alsowiyan, Abdulrahman K Almutairi, Amal Mohamed, Abdulrahman M Almutiq

**Affiliations:** 1 Surgery, King Fahad Specialist Hospital, Buraydah, SAU; 2 General Surgery, King Fahad Specialist Hospital, Buraydah, SAU; 3 College of Medicine, Qassim University, Buraydah, SAU; 4 College of Medicine, University of Khartoum, Khartoum, SDN

**Keywords:** buraydah, gallstones, gallstones complication, ksa, seasonal variation

## Abstract

Introduction

Seasonal variation is a common factor in the development of gastrointestinal conditions, including gallstone complications such as chronic cholecystitis, acute cholecystitis (AC), and acute biliary pancreatitis. The aim of this study is to evaluate the impact of seasonal temperature variation on the incidence of gallstone complications in adult patients admitted to King Fahad Specialist Hospital (KFSH) in Buraydah, Saudi Arabia.

Methods

This retrospective study analyzed 1,719 complete medical records of adult patients (>15 years) undergoing emergent or elective cholecystectomy at KFSH, Buraydah, Saudi Arabia, for gallstone-related indications (acute biliary pancreatitis, AC, common bile duct (CBD) or biliary obstruction, chronic cholecystitis, first-time diagnosed uncomplicated gallstones). Patients <15 years and those with incomplete records, non-gallstone, or rare gallstone complications (e.g., fistula, polyps, adenomyomatosis, Lemmel syndrome, Mirizzi syndrome, malignancy) were excluded. Diagnoses were standardized via validated ultrasound reports, and seasons were assigned based on the latest ultrasound diagnosis. Data were processed using IBM SPSS Statistics for Windows, Version 27.0 (Released 2020; IBM Corp., Armonk, New York, United States).

Results

The study revealed that the summer season had the highest frequency of first-time diagnosed gallstones with and without complications (n=716, 41.7%). Overall, the prevalence of gallstone complications was found in 528 patients (30.7%). The most common gallstone complications were AC (49.2%, 260 cases), followed by CBD or biliary tree obstruction (31.4%, 166 cases). Additionally, age (adjusted OR (AOR)=2.092; 95%CI=1.214- 3.603; p=0.008) and gender (AOR=1.590; 95%CI=1.280- 1.976; p <0.001) were identified as significant independent predictors of gallstone complications, with male patients and those aged 65 years and above being more likely to develop gallstone complications

Conclusion

The study demonstrated a trend toward increased gallstone complications during the summer season among adult patients in Buraydah, Saudi Arabia; however, this seasonal variation was not statistically significant after multivariate analysis (p = 0.318). Male sex and age ≥65 years were identified as significant predictors. These findings support targeted awareness efforts focused on high-risk populations.

## Introduction

Gallstones can be dangerous and can cause several health problems, one of which is acute cholecystitis (AC). When gallstones develop into AC, they create an obstruction of the cystic duct [1]. As a result of this obstruction, the gallbladder swells in a manner to form the condition. AC is a serious condition that causes many people to be admitted to hospitals. Prevalence of AC varies from one country and region to another, but it tends to be more prevalent in the developed world as compared to developing countries [2].

About 20-30% of people with gallstones are symptomatic [3], while 1-3% of patients with symptomatic gallstone have complications annually [4]. The prevalence of gallstones and AC is greatest in highly developed areas such as Europe and North America [3,4]. The likelihood of acquiring AC is affected by multiple factors, including age, gender, lifestyle, sickle cell disease, and type 2 diabetes [1]. Research indicates that the incidence of AC exhibits seasonal trends, with certain times of the year experiencing a more pronounced increase. This variation has been associated with multiple causes, including environmental influences. For example, extreme changes in temperature, humidity, and dietary habits have been cited as having the capacity to influence the occurrence of cholecystitis [5]. This is because these extreme changes can affect the functioning of the gallbladder. The association between seasonal variations and the incidence of AC was reported by Taib et al., who indicated that there was a spike in the cases of AC during the summer, which was attributable to increased dehydration, cholesterol saturation in bile, and fat intake [6].

In Saudi Arabia, AC is highly prevalent, with Alishi et al. reporting an 8.6% prevalence of the disease [7]. The relatively high prevalence can be expected, especially given that the incidence of gallstones in the country and the whole Middle East and North Africa (MENA) region is also high. One major reason that has been linked to the high incidence of gallstones in Saudi Arabia is the fact that people ordinarily eat high-fat and low-fiber diets [8]. Further, the weather in the Middle East has been cited as a possible cause of a high rate of progression of gallstones to AC [9]. Considering that Saudi Arabia is susceptible to most risk factors, including extreme temperature fluctuations, it is important to investigate and understand the potential of seasonal variations in influencing the onset of AC. Therefore, the objective of this investigation is to assess the impact of seasonal variations on the onset of gallstone complications among adult patients at the King Fahad Specialist Hospital in Buraydah, Saudi Arabia.

## Materials and methods

Study design and population

This was an analytic study that employed a retrospective chart review, reviewing the medical records and operation room (OR) lists of patients who underwent cholecystectomy in King Fahad Specialist Hospital, the largest hospital in Buraydah, the capital of the Al-Qassim region, from January 1, 2018, to November 30, 2024. The study was approved by the Regional Research Ethics Committee, Qassim Province (registration number: 607-46-9551) and conducted in accordance with ethical guidelines, ensuring patient confidentiality and data protection. As this study involved retrospective data, no direct patient contact was required, and patient identifiers were anonymized to maintain privacy.

Patients who had cholecystectomy due to gallstones (cholelithiasis) or gallstone-related complications (AC, chronic cholecystitis, CBD or biliary tree obstruction, or biliary pancreatitis) were included in this study. Patients with incomplete medical records or who underwent cholecystectomy for a non-gallstone-related indication were excluded from the study. The sample size was determined based on data availability from hospital records.

Data collection

Data were gathered from both electronic and paper-based hospital records, extracting relevant details such as patient demographics (age, gender, comorbidities, etc.), indication of cholecystectomy (AC, persistent biliary colic, or other complications), and the monthly distribution of the diagnoses to evaluate seasonal variations and trends. Additionally, data on the type of intervention performed, the length of hospital stay, post-treatment outcomes, and recurrence of symptoms were gathered to provide comprehensive insights into patient care and recovery.

Seasonal definitions used in the study

For the purposes of this study, the seasons were defined as follows: Summer: May, June, July, August, September; Winter: December, January, February; Fall: October, November; Spring: March, April.

Data management and analysis

The gathered data were inserted and cleaned using Microsoft Excel (Microsoft Corporation, Redmond, Washington, United States) prior to statistical analysis with IBM SPSS Statistics for Windows, Version 27.0 (Released 2020; IBM Corp., Armonk, New York, United States). Descriptive statistics, encompassing frequencies and percentages, were employed to encapsulate patient characteristics and first-time diagnosis trends. Chi-square and Fisher’s exact tests were employed to evaluate the correlation between diagnosis rates and seasonal fluctuations. Univariate logistic regression and multivariate logistic regression were employed as well. A p-value of less than 0.05 was deemed statistically significant.

## Results

A total of 1719 patients were included in the study. Sociodemographic characteristics of the patients (Table 1) show that the majority of the patients were female (n=1169, 68.0%) with a considerable proportion of the patients with a BMI of 25.0-29.9 (n=712, 41.4%). Regarding the age groups, the patients were almost equally distributed across the various age categories, with no considerable skew toward any particular group. 

**Table 1:** Sociodemographic information of the patients

Characteristic	Category	Frequency (Percentage)
Age Group (years)	15-25 years	233 (13.6%)
	26-30	276 (16.1%)
	31-35	294 (17.1%)
	36-40	248 (14.4%)
	41-45	188 (10.9%)
	46-50	181 (10.5%)
	51-55	129 (7.5%)
	56-60	65 (3.8%)
	61-65	50 (2.9%)
	Above 65 years	55 (3.8%)
Gender	Female	1169 (68.0%)
	Male	550 (32.0%)
Body Mass Index (kg/m^2^)	<18.5	9 (0.5%)
	18.5-24.9	494 (28.7%)
	25.0-29.9	712 (41.4%)
	30.0-34.9	332 (19.4%)
	35 or above	172 (10.0%)

The clinical characteristics of the patients are given in Table 2. A total of 260 patients (15.1%) were diagnosed with AC, followed by CBD or biliary tree obstruction (n=166, 9.7%), acute biliary pancreatitis (60, 3.5%), and chronic cholecystitis (42, 2.4%). The majority had no complications (n=1191, 69.3%).

**Table 2:** Clinical characteristics of the patients

Characteristics	Category	Frequency (Percentage)
Diagnosis	Acute Biliary Pancreatitis	60 (3.5%)
	Acute Cholecystitis	260 (15.1%)
	CBD or Biliary Tree Obstruction	166 (9.7%)
	Chronic Cholecystitis	42 (2.4%)
	Gallstones (Cholelithiasis) “Without complication”	1191 (69.3%)
Date of procedure	>1 year from the diagnosis	58 (3.4%)
	6-12 months from the diagnosis	156 (9.0%)
	Within 6 months of the diagnosis	1505 (87.6%)
Type of procedure	Elective	1246 (72.5%)
	Emergency	473 (27.5%)

**Figure 1:**
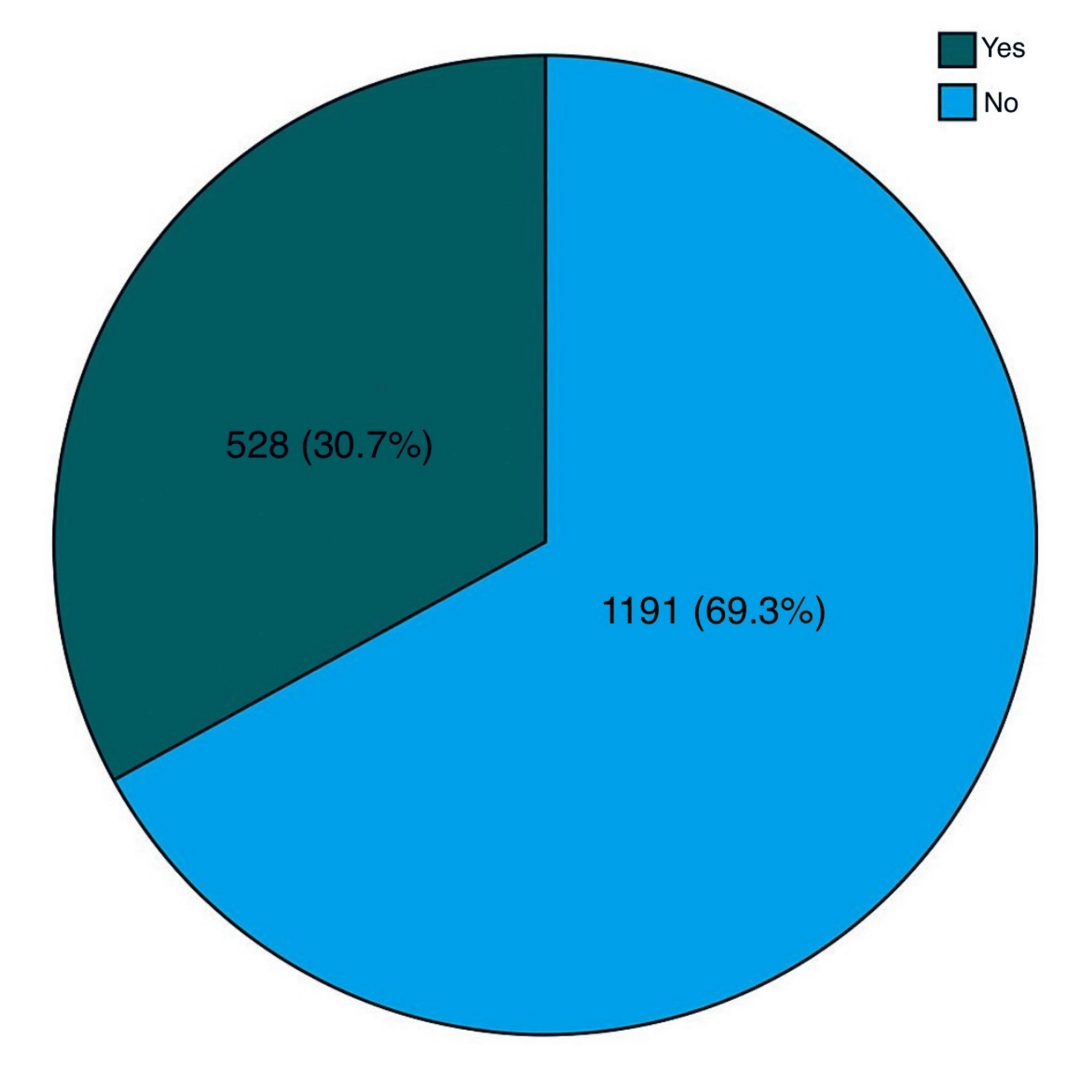
Prevalence of gallstone complications (N=1719)

Of the 1719 patients with gallstones, one-third of 528 patients (30.7%) had complications, while 1191 patients (69.3%) had no complications, as shown in Figure 1.

There was a significant association between age groups and gender with gallstone complications (p-values 0.023 and p<0.001), respectively (Table 3). However, no statistically significant association was found between BMI and gallstone complications (p > 0.05).

**Table 3:** The association between age, gender, and BMI with gallstone complications * Significant at p<0.05 level.

Charateristics	Category	No Gallstone Complications, n (%)	Presence of Gallstone Complications, n (%)	p-value
Age group (years)	15-25	171 (73.4%)	62 (26.6%)	0.023*
	26-30	179 (64.9%)	97 (35.1%)	
	31-35	209 (71.1%)	85 (28.9%)	
	36-40	168 (67.7%)	80 (32.3%)	
	41-45	138 (73.4%)	50 (26.6%)	
	46-50	138 (73.4%)	50 (27.6%)	
	51-55	93 (72.1%)	36 (27.9%)	
	56-60	39 (60.0%)	26 (40.0%)	
	61-65	35 (70.0%)	15 (30.0%)	
	Above 65 years	28 (50.9%)	27 (49.1%)	
Gender	Female	849 (72.6%)	320 (27.4%)	<0.001*
	Male	342 (62.2%)	208 (37.8%)	
Body Mass Index (Kg/m^2^)	<18.5	6 (66.7%)	3 (33.3%)	0.888
	18.5-24.9	340 (68.8%)	154 (31.2%)	
	25.0-29.9	489 (68.7%)	223 (31.3%)	
	30.0-34.9	238 (71.7%)	94 (28.3%)	
	35 or above	118 (68.6%)	54 (31.4%)	

**Figure 2:**
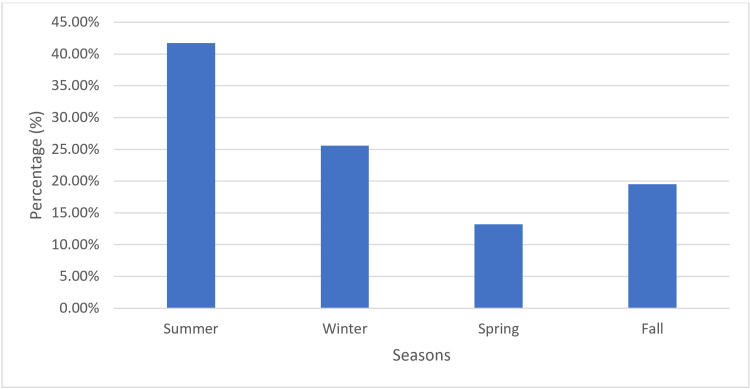
Seasonal frequency of gallstone or gallstone complications

Figure 2 illustrates the seasonal frequency of gallstone or gallstone complications. The summer season (May, June, July, August, September) had the highest incidence, with 716 cases (41.7%), followed by winter (December, January, February) with 440 cases (25.6%), fall (October, November) with 335 cases (19.5%), and spring (March, April) with 228 cases (13.2%).

The number of first-time diagnoses and gallstone complications each month is given in Table 4. The study observed that the highest number of first-time diagnoses of gallstones and most complications occurred towards the end of summer/beginning of fall.

**Table 4:** Distribution of total first-time diagnoses and gallstone complications in each month of the year Data given as frequency (percentage)

Month	Acute Biliary Pancreatitis (n=60)	Acute Cholecystitis (n=260)	CBD or Biliary Tree Obstruction (n=166)	Chronic Cholecystitis (n=42)	Gallstones Cholelithiasis “Without Complication” (n=1191)	Monthly total first-time diagnoses with and without complications
January	6 (10.0%)	20 (7.7%)	21 (12.7%)	4 (9.5%)	106 (8.9%)	157 (9.1%)
February	7 (11.7%)	18 (6.9%)	14 (8.4%)	3 (7.1%)	81 (6.8%)	123 (7.2%)
March	5 (8.3%)	24 (9.2%)	12 (7.2%)	2 (4.8%)	91 (7.6%)	134 (7.8%)
April	6 (10.0%)	24 (9.2%)	8 (4.8%)	2 (4.8%)	54 (4.5%)	94 (5.5%)
May	0 (0.0%)	26 (10.0%)	13 (7.8%)	1 (2.4%)	73 (6.1%)	113 (6.6%)
June	5 (8.3%)	10 (3.8%)	21 (12.7%)	0 (0.0%)	87 (7.3%)	123 (7.2%)
July	3 (5.0%)	28 (10.3%)	10 (6.0%)	2 (4.8%)	105 (8.8%)	148 (8.6%)
August	9 (15.0%)	17 (6.5%)	15 (9.0%)	9 (21.4%)	93 (7.8%)	143 (8.3%)
September	6 (10.0%)	23 (8.8%)	18 (10.8%)	5 (11.9%)	137 (11.5%)	189 (11.0%)
October	6 (10.0%)	27 (10.4%)	12 (7.2%)	5 (11.9%)	131 (11.0%)	181 (10.5%)
November	1 (1.7%)	18 (6.9%)	12 (7.2%)	7 (16.7%)	116 (9.7%)	154 (9.0%)
December	6 (10.0%)	25 (9.6%)	10 (6.0%)	2 (4.8%)	117 (9.8%)	160 (9.3%)

The number of first-time gallstone diagnoses was the highest in summer (n=716, 41.7%), followed by winter (n=440, 25.6%), fall (n=335, 19.5%), and spring (n=228, 13.3%) (Table 5). It was observed that most gallstone complications were diagnosed during the summer season, including CBD or biliary tree obstruction (n=77, 46.4%), chronic cholecystitis (n=17, 40.5%), and AC (n=104, 40.0%), which was most prevalent in the month of October (n=27, 10.4%) and July (n=28, 10.3%). Additionally, most of the gallstone complications were observed during the summer (221, 41.9%). However, these differences were not statistically significant (p>0.05). Most of the surgeries were performed during the summer (716, 41.7%), followed by winter (440, 25.6%), fall (335, 19.5%), with the fewest performed in the spring (228, 13.3%). Additionally, there was a significantly higher proportion of emergency surgeries compared to elective surgeries in the summer, winter, and spring (p=0.018).

**Table 5:** Relationship of the number of first time diagnosis of gallstone or gallstone complications with seasons of the year * Significant at p<0.05 level.

Variables	Category	Summer	Winter	Spring	Fall	Total	p-value
First-time Diagnosis	Acute Biliary Pancreatitis	23 (38.3%)	19 (31.7%)	11 (18.3%)	7 (11.7%)	60 (100.0%)	0.107
	Acute Cholecystitis	104 (40.0%)	63 (24.2%)	48 (18.5%)	45 (17.5%)	260 (100.0%)	
	CBD or Biliary Tree Obstruction	77 (46.4%)	45 (27.1%)	20 (12.0%)	24 (14.5%)	166 (100.0%)	
	Chronic Cholecystitis	17 (40.5%)	9 (21.4%)	4 (9.5%)	12 (28.6%)	42 (100.0%)	
	Gallstones Cholelithiasis “Without Complication”	495 (41.6%)	304 (25.5%)	145 (12.2%)	247 (20.7%)	1191 (100.0%)	
	Total	716 (41.7%)	440 (25.6%)	228 (13.3%)	335 (19.5%)	1719 (100.0%)	
Complications	Yes	221 (41.9%)	136 (25.8%)	83 (15.7%)	88 (16.7%)	528 (100.0%)	0.086
	No	495 (41.6%)	304 (25.5%)	145 (12.2%)	247 (20.7%)	1191 (100.0%)	
	Total	716 (41.7%)	440 (25.6%)	228 (13.3%)	335 (19.5%)	1719 (100.0%)	
Type of Procedure	Elective	509 (40.9%)	316 (25.4%)	156 (12.5%)	265 (21.3%)	1246 (100.0%)	0.018*
	Emergency	207 (43.8%)	124 (26.2%)	72 (15.2%)	70 (14.8%)	473 (100.0%)	
	Total	716 (41.7%)	440 (25.6%)	228 (13.3%)	335 (19.5%)	1719 (100.0%)	

Most surgeries were elective, with 1,246 elective surgeries (72.5%) compared to 473 emergency surgeries (27.5%) (Table 6). A majority of cases of acute biliary pancreatitis (n=42, 70.0%) and AC (n=151, 58.1%) required emergency surgery.

**Table 6:** Association between diagnosis and the type of surgery * Significant at p<0.05 level.

Variables	Category	Elective, n (%)	Emergency, n (%)	Total, n (%)	p-value
Diagnosis	Acute Biliary Pancreatitis	18 (30.0%)	42 (70.0%)	60 (100.0%)	<0.001*
	Acute Cholecystitis	109 (41.9%)	151 (58.1%)	260 (100.0%)	
	CBD or Biliary Tree Obstruction	88 (53.0%)	78 (47.0%)	166 (100.0%)	
	Chronic Cholecystitis	32 (76.2%)	10 (23.8%)	42 (100.0%)	
	Gallstones Cholelithiasis “Without Complication”	999 (83.9%)	192 (16.1%)	1191 (100.0%)	
Total		1246 (72.5%)	473 (27.5%)	1719 (100.0%)	

Univariate regression analysis (Table 7) revealed that age (adjusted OR (AOR) = 2.238; 95%CI = 1.306-3.837; p = 0.003) and gender (AOR = 1.614; 95%CI = 1.301-2.001; p < 0.001) were significant predictors for the onset of gallstone complications. It was observed that being a male was associated with a 1.614 times higher odds of developing gallstone complications. Additionally, individuals aged 65 years and above had 2.238 times higher odds of developing gallstone complications. In contrast, BMI (AOR = 0.975; 95%CI = 0.874-1.087; p = 0.643) and seasons (AOR = 0.963; 95%CI = 0.881-1.054; p = 0.415) were not significant predictors.

**Table 7:** Univariate logistic regression to predict the onset of gallstone complications AOR: adjusted odds ratio; CI: confidence interval ** Significant at p<0.05 level

Factor	AOR	95% CI	P-value
Gender	1.614	1.301 – 2.001	<0.001**
Age	2.238	1.306 – 3.837	0.003**
Body Mass Index (BMI)	0.975	0.874 – 1.087	0.643
Seasons	0.881	0.881 – 1.054	0.415

Multivariate regression analysis (Table 8) revealed that age (AOR = 2.092; 95%CI = 1.214-3.603; p = 0.008) and gender (AOR = 1.590; 95%CI = 1.280-1.976; p < 0.001) remained significant predictors of the onset of gallstone complications after adjusting for other variables. Specifically, male patients and individuals aged 65 years and above had a higher likelihood of developing gallstone complications. In contrast, BMI (AOR = 0.998; 95%CI = 0.894-1.115; p = 0.974) and seasons (AOR = 0.955; 95%CI = 0.872-1.046; p = 0.318) did not significantly alter the probability of developing complications after controlling for other factors.

**Table 8:** Multivariate logistic regression to predict the onset of gallstone complications AOR: adjusted odds ratio; CI: confidence interval ** Significant at p<0.05 level

Factor	AOR	95% CI	P-value
Gender (male)	1.590	1.280 – 1.976	<0.001**
Age (≥65 years)	2.092	1.214 – 3.603	0.008**
Body Mass Index (BMI)	0.998	0.894 – 1.115	0.974
Seasons	0.955	0.872 – 1.046	0.318

## Discussion

The incidence of gallbladder inflammation due to gallstones in patients can be attributed to a number of factors, including lifestyle, demographics, health conditions of patients, as well as seasonal variations, including changes in environmental factors, diet, and temperature, which may influence the development of gallstone complications [10,11]. Given the extreme climatic conditions in Saudi Arabia with mild winters and hot summers, this study aimed to assess how the seasonal variations influence the onset of gallstone complications among adult patients at KFSH in Buraydah, Saudi Arabia.

The study revealed that nearly one-third (30.7%) of the patients had gallstone complications, highlighting a considerable prevalence of the condition, which may be attributed to various risk factors, including the interaction of environmental and genetic factors, diabetes, pregnancy, aging, or obesity [12]. The common gallstone complications reported in the current study included AC (n=260, 49.2%) followed by CBD or biliary tree obstruction (n=166, 31.4%). The finding is consistent with studies conducted by Wrenn et al. [13] and Sigmon et al. [14], which reported that AC was the commonly diagnosed condition in over 20% of the histopathology examination of the gallbladder specimen.

The current study revealed a significant association between gender and gallstone complications, with a considerably higher proportion of male patients (37.8%) experiencing complications compared to female patients (27.4%). Additionally, male patients were found to have a higher likelihood of developing gallstone complications than females (AOR = 1.590; 95%CI = 1.280-1.976; p < 0.001). This could be attributed to gender differences in lifestyle and dietary habits, as the majority of men in Saudi Arabia tend to have less controlled diets, often consuming processed foods and diets high in sugar and fats, particularly during the summer months and events like Ramadan. These dietary habits may increase their risk of developing gallstone complications.

The study observed that patients aged 65 years and above had a significantly higher prevalence of gallstone complications (49.1%) compared to other age groups. Additionally, they had a higher likelihood of developing gallstone complications compared to younger patients (AOR = 2.092; 95%CI = 1.214-3.603; p = 0.008). This can be attributed to changes in the composition of bile in the gallbladder, resulting in the formation of gallstones with time as the patient ages.

The study found summer season to have the highest frequency of gallstone diagnoses with and without gallstone complications (n=716, 41.7%); which is consistent with Zangbar et al.’s observation that gallstone complications, including AC, occur more frequently in summer than in winter [15]. Similar observations were made in a Saudi study conducted by Hosseini et al. [16] and in Taiwan by Liu et al. [17], which reported a notable increase in AC in the summer. Furthermore, the present study observed a significantly higher number of first-time diagnoses of gallstones with AC in the summer, particularly in the months of July and October. This can be attributed to the extreme summer temperatures experienced in Saudi Arabia in the month of July, which can cause dehydration in patients, underscoring the importance of individuals keeping hydrated by drinking adequate water in order to prevent the risk of developing gallstone complications throughout the summer season.

A major limitation of this analysis is the reliance on retrospective data from medical records, which could have potentially led to incomplete or missing critical information. Additionally, excluding patients undergoing non-gallstone cholecystectomy introduces potential selection bias. The lack of adjustment for patient comorbidities could further confound findings, especially concerning age-related risk assessments. These limitations may present significant hindrances to achieving comprehensive insights into the risk factors associated with gallstone complications.

## Conclusions

The study identified age and gender as significant independent predictors of gallstone complications among adults in Buraydah, Saudi Arabia, with higher rates observed in men and those aged 65 years and above. Although a seasonal increase was noted during summer, this variation did not retain statistical significance after adjustment. These findings highlight the importance of focusing public education and healthcare strategies on the more robust predictors to improve patient outcomes.
